# Long-Run Savings and Investment Strategy Optimization

**DOI:** 10.1155/2014/510531

**Published:** 2014-02-23

**Authors:** Russell Gerrard, Montserrat Guillén, Jens Perch Nielsen, Ana M. Pérez-Marín

**Affiliations:** ^1^Cass Business School, City University London, 106 Bunhill Row, London EC1Y 8TZ, UK; ^2^Department of Econometrics, Riskcenter-IREA, University of Barcelona, Avenue Diagonal 690, 08034 Barcelona, Spain

## Abstract

We focus on automatic strategies to optimize life cycle savings and investment. Classical optimal savings theory establishes that, given the level of risk aversion, a saver would keep the same relative amount invested in risky assets at any given time. We show that, when optimizing lifecycle investment, performance and risk assessment have to take into account the investor's risk aversion and the maximum amount the investor could lose, simultaneously. When risk aversion and maximum possible loss are considered jointly, an optimal savings strategy is obtained, which follows from constant rather than relative absolute risk aversion. This result is fundamental to prove that if risk aversion and the maximum possible loss are both high, then holding a constant amount invested in the risky asset is optimal for a standard lifetime saving/pension process and outperforms some other simple strategies. Performance comparisons are based on downside risk-adjusted equivalence that is used in our illustration.

## 1. Introduction

We study long term investment strategies characterised by a first period of savings followed by a period of consumption after retirement. This is the most common form of optimization problem that is faced by most citizens, who seek a way to build up a pension payment stream that provides a balance between risk and reward. Most investors request downside protection and desire upside potential.

There are two competing magnitudes related to risk in classical optimality theory when studying lifetime savings and retirement investments. One is the maximum amount the saver is allowing to lose during his life cycle. The other is his risk aversion, a parameter that is linked to the power of a power utility function. However, risk aversion and maximum possible loss have not been studied together. Most articles focus on one and consider the other parameter as fixed. When looking at the distribution of terminal wealth, our results show that those two parameters should not be considered separately when optimizing long-term expected returns, while controlling for downside risk.

In this paper, we use the risk adjusted ad hoc performance measure recently developed in [1, 2] to evaluate the consequences of choosing a given combination of maximum loss and risk aversion. We study optimality of a constant absolute risk aversion investment strategy that differs slightly from but is reminiscent of standard Constant Proportion Portfolio Insurance (CPPI), which was introduced by [3] (see also, [4–6]). It is unsurprising that combining a high maximum possible long-term loss with a high risk aversion, that is, a lower power in the power utility function, will be superior in the long run to the risk-adjusted equivalent with a lower long-term maximum possible loss and a lower risk aversion, that is, higher power in the utility function. However, the first combination is a good deal better than the second when studying the distribution of terminal wealth, leading us to further investigations in this direction. We take this point of view to its logical extreme and let the maximum loss go to infinity at the same time as the power in the utility function goes to zero, which means that we also raise risk aversion to infinity. Our limit behaviour is carried out in such a way that the limit of power utility functions is also a utility function, which happens to be the exponential utility and which gives a fixed constant absolute risk aversion. It turns out that both the optimal strategy in the constant relative risk aversion and the performance measures converge smoothly to the equivalent entities derived directly from the exponential utility.

Our contribution is to establish the link between risk aversion and maximum possible loss in some classical utility function optimization. We also show that performance analysis in terms of downside protection and risk/return trade-off enhances the understanding of lifetime investment strategies.

In Section 2, we provide some background and notation. In Section 3, we introduce the market model and the optimisation problem which will be used to illustrate the results on power utility functions. The optimal strategy prescribes an initial investment in the risky asset proportional to the ratio between maximum possible loss and risk aversion, which is a principal reason for keeping this quantity fixed when passing to the limit. We demonstrate that the optimal strategy for the generalized shifted utility that is defined when wealth can be negative (as is permitted, up to a certain maximum loss) converges to a limiting strategy, and that is indeed the optimal strategy under the limiting utility function.  Section 4 discusses the risk and performance measures of lifetime investment plans and the approach to the comparison of different strategies. We use risk measure tools that are generally accepted in this context.

Our focus here is the expected shortfall risk measures [7, 8] such as downside value at risk [9, 10] and tail conditional expectation [11–13] which are widely accepted in financial economics. The probability of a lifetime ruin is already studied by [14], as well as optimal portfolio selection terminal wealth problems (see [15]). Lifetime ruin has received increased attention (see, [16]).

We use simulations to evaluate the expected shortfall for “benchmark” strategies; that is, those which prescribe that a constant proportion of the wealth should be invested in the risky asset. We call these “benchmark” strategies because this type of strategies is frequently recommended by investment advisors [17–19]. Similar questions have been investigated by many authors [20–29]. More recently, reference [30] contributed to the analysis of investment strategies that meet certain targets (see also [31]). In our presentation, we fix a constant proportion and then we calculate the value of the maximum possible loss such that the generalized power utility-optimal strategy produces the same expected shortfall and proceed to compare the two strategies in terms of the internal rate of return (IRR). We also investigate whether it is possible to find a benchmark strategy which can give the same IRR as the shifted power utility-optimal strategy, or special CPPI. The discussion of the results which takes place in Section 5 is followed by Section 6 which concludes.

## 2. Background and Preliminaries

It is well documented in the finance literature that, under very general conditions, the power utility function leads to constant relative risk exposure; that is, a constant proportion of the wealth is invested in risky assets, with the remainder in risk-free assets. An investment strategy that maximizes the power utility function is called constant relative risk aversion (CRRA). See [32] for one of the more compact and general introductions. So, in practice, an investor maximizing power utility would invest a fixed proportion of his wealth in the stock market and the remainder in bonds. He would revise his positions about once a year, as the stock prices and returns are known. Then gains would be accumulated to previous year's wealth. The outcomes are such that each year's wealth distribution depends on the previous year's outcome. So, yearly gains (or losses) matter a lot to the next period investment balance between risk-free and risky assets.

Alternatively, another investor may prefer to fix an amount of his wealth to be invested in the stock market. That would correspond to a strategy called constant absolute risk aversion (CARA). In other words, this investor would take a constant amount and would invest that in the risky assets, keeping the rest of his wealth in risk-free investment. Generally, after one year, no matter what the gains are, he would again invest the same constant amount in stocks. In a pure CPPI strategy, the investor would have defined a floor and a multiplier and then his exposure to the risky assets would be a constant multiple of the cushion, which is the excess of wealth over the floor. Our investor however does not establish a floor nor a cushion and does not need to look into yearly gains (or losses) to tune the next period's investment balance. This investor does not have a downside protection; however, we will require for him an immense risk aversion. Then, we will find out that he can outperform the risk-adjusted equivalent strategy followed by the first investor.

Investment strategies resulting from a constant relative risk exposure and from a constant absolute risk exposure correspond to the maximization of utility functions that are related. We provide the theory to show that investors choosing a constant absolute risk exposure can outperform those that use a constant relative risk exposure approach. Indeed, those two strategies correspond to the optimization of two different utility functions, that can be written in such a way that the constant absolute risk exposure is obtained as the optimum of the utility function which is the limit of the utility that has the optimum in the constant relative risk exposure.

The practical consequences of this general theory when it comes to analysing the distribution of final wealth and optimal long-term savings have not received much coverage in the literature, but recently some papers have started to look into stochastic dominance, downside risk and performance evaluation of portfolio insurance strategies. Unfortunately, these contributions look closely into most popular products like stop-loss, synthetic put, and constant proportion portfolio insurance techniques (see, [33–36]). These strategies produce wealth processes that depend on the previous year's wealth, which are known to be suboptimal in the usual market hypothesis [37–39].

### 2.1. Time Framework and Terminal Wealth Distribution

In our paper, we study long-term investment, because we think that many savings investors consider optimal investment strategies on the basis of having a long period of savings and then a retirement phase when they need a stream of income for consumption or a pension. So, we focus on long periods of at least sixty years.

Lifetime savings are difficult to study because each person has a particular cash flow pattern. We will consider a simple payment stream, since we will not assume inflation, then we can assume that saving/consumption payments are constant and periodical during a life cycle. Typically, a pension plan establishes a period of many years of savings, for instance, from age 35 to age 65 (retirement age). Then, after retirement, the saver needs to withdraw money from his investment account on a regular basis for about another period of 30 years, until time *T*. This setting is similar to the time framework chosen for other authors who address retirement income (see [40]). Moreover, to simplify our setting, we do not consider mortality uncertainty (A recent discussion on mortality risk transfer can be found in [41].), as our time horizon *T* is fixed.

An obvious question is to find an optimal way to invest the initial stream of payments that will accumulate wealth in the savings period and also to keep the remaining investment during the withdrawal time. Any investor would prefer a strategy that will ultimately provide him with the highest return. However, the uncertainty about the evolution of the returns makes prediction impossible. We can only work with assumptions on the returns process and study the wealth distribution at time *T*. The wealth distribution is the set of possible returns together with the odds to achieve them. In this paper, instead of limiting our study to fixed terminal wealth distribution we will rather fix its downside risk level. There is a risk that the investor does not have enough resources to be able to withdraw a pension during retirement. Many investors could be enormously averse to the risk of ruin, or even to the risk that wealth at time *T* falls below a certain level if they are interested in leaving a bequest to their heirs. The utility function captures the preference, from the investor's point of view, of the final wealth outcome (see, e.g., [6, 42, 43]).

Investment planning is a central topic, for pension funds [44, 45], for mutual funds and also for individuals. The model by [44] uses a multiperiod stochastic linear programming framework with a flexible number of time periods of varying length. Not many researchers deal with the whole lifespan investment horizon. The work by [46] looks at similar problems, but they rather focus on the fluctuations of income streams and on portfolio choice [47] rather than on proportion versus constant wealth in the bond versus stock combination. Our study uses a timing for lifetime savings similar to the one proposed by [48] or the one used by [49] for illustrative purposes. Both establish about thirty years of savings followed by about thirty more years of retirement.

Performance evaluation of portfolio insurance strategies using stochastic dominance criteria was studied by [33] who found that in typical CPPI strategies, a higher CPPI multiple enhances the upward potential but harms the protection level. They also indicated that choosing a different floor value or relaxing the rebalancing discipline substantially harms the strategies' performance. Our approach is similar to theirs but compares strategies that vary in terms of the combination of risk aversion and maximum possible loss.

### 2.2. The Power Utility Function and the Optimisation of Lifelong Savings

Utility functions are widely used by financial economists because they measure the “value” of wealth to investors. Concave utility functions are preferred because they reflect risk-aversion; that is, the utility of potential wealth increase does not compensate the risk or the utility of wealth loss.

In the simplest possible case we define the power utility function by

(1) $$\begin{array}{c} u_{\gamma }\left(c\right)=\gamma^{-1}c^{\gamma }, \end{array}$$

where *c* and 1 − *γ* are positive real numbers. The value of 1 − *γ* is known as relative risk aversion or simply risk aversion. The larger it is, the larger is risk aversion and the flatter is the utility shape as *c* increases. Central features of utility functions are their relative and absolute risk aversions (see, [50]). Note that this utility is only defined for positive wealth and thus losses or negative wealth are not allowed.

In this case the relative risk aversion is given by

(2) $$\begin{array}{c} R\left(c\right)=-c\frac{u_{\gamma }^{\prime \prime }\left(c\right)}{u_{\gamma }^{\prime }\left(c\right)}=1-\gamma , \end{array}$$

and the absolute risk aversion is

(3) $$\begin{array}{c} A\left(c\right)=-\frac{u_{\gamma }^{\prime \prime }\left(c\right)}{u_{\gamma }^{\prime }\left(c\right)}=\frac{1-\gamma }{c}. \end{array}$$

In a power utility function, there is a constant relative risk aversion (CRRA), while absolute risk aversion decreases when *c* increases.

Now let us introduce a second parameter *K* so that

(4) $$\begin{array}{c} \;\;u_{\gamma ,K}\left(c\right)=\gamma^{-1}{\left(c+K\right)}^{\gamma }, \end{array}$$

where *c* > −*K* and 1 − *γ* > 0. Now, we can accept having negative wealth and the utility is defined in the interval (−*K*, 0] as well as in the positive real line, as the standard power utility function. Then we have that relative risk aversion is

(5) $$\begin{array}{c} R\left(c\right)=-c\frac{u_{\gamma ,K}^{\prime \prime }\left(c\right)}{u_{\gamma ,K}^{\prime }\left(c\right)}=\left(1-\gamma \right)\frac{c}{c+K}, \end{array}$$

and absolute risk aversion is

(6) $$\begin{array}{c} A\left(c\right)=-\frac{u_{\gamma ,K}^{\prime \prime }\left(c\right)}{u_{\gamma ,K}^{\prime }\left(c\right)}=\left(1-\gamma \right)\frac{1}{c+K}. \end{array}$$

For this extended power utility, neither CRRA nor CARA hold. Under this setting that allows for the existence of losses, risk aversion depends on the two parameters *γ* and *K*.

### 2.3. Limiting Utility Function

One of the features of interest of the present paper is the investigation of the limiting case, where *K* → *∞* and *γ* → −*∞* in such a way that *K*/(1 − *γ*) is equal to a constant, Λ. This corresponds to a balanced situation, where an increasing maximum possible allowed loss *K* and an increasing risk aversion (1 − *γ*) tend to an equilibrium in the limit.

Although the utility functions *u*_*γ*,*K*_ do not converge in a formal sense, we may make use of the fact that, if one utility function is a linear function of another, then the maximization of the two utility functions will give rise to identical strategies. Let us then renormalize *u*_*γ*,*K*_(*c*) in such a way that *u*_*γ*,*K*_′(0) = 1. This produces a new utility function:

(7) $$\begin{array}{c} {\overset{-}{u}}_{\gamma ,K}\left(c\right)=\frac{1}{\gamma K^{\gamma -1}}{\left(c+K\right)}^{\gamma }. \end{array}$$

Now, we set *K* = Λ(1 − *γ*) to obtain

(8) $$\begin{array}{c} {\overset{-}{u}}_{\gamma ,K}\left(c\right)=\frac{\Lambda \left(1-\gamma \right)}{\gamma }{\left(1+\frac{c}{\Lambda (1-\gamma )}\right)}^{\gamma }. \end{array}$$

As *γ* → −*∞*, the limit of this is

(9) $$\begin{array}{c} \overset{-}{u}\left(c\right)=-\Lambda e^{-c/\Lambda }. \end{array}$$

The utility function $\overset{-}{u}$ produced by this limiting procedure has constant absolute risk aversion, $A(c)=-{\overset{-}{u}}^{\prime \prime }(c)/{\overset{-}{u}}^{\prime }(c)=\Lambda^{-1}$. So, an investment strategy that maximizes this utility has the constant absolute risk aversion property.

This result shows that there is a continuous and smooth transition from the power utility function with constant relative risk aversion to the constant absolute risk aversion case. Below, we will investigate this transition by employing a risk measure to determine the behaviour of the process under optimal control when utility function ${\overset{-}{u}}_{\gamma ,K}$ is used and to take the limit of this as *γ* → −*∞*. We will finally compare the limiting results with those derived directly in the constant absolute risk aversion case.

We will see that having an adequate combination of huge risk aversion and enormous maximum loss under the power utility function leads to the constant absolute utility function of the exponential utility in (9).

## 3. The Optimisation Problem

In this paper, we address the problem of choosing a strategy for the selection of an asset mix in such a way as to maximize the expected utility of terminal value at time *T* of an investment consisting of a sequence of annual premiums of size *a* paid into an account at times 0, 1,…, *T*/2 − 1, followed by a sequence of annual withdrawals of benefits, also of size *a*, at times *T*/2, *T*/2 + 1, …, *T* − 1. This is a continuous time investigation, in the sense that the investment portfolio can be adjusted at any time, not just at the times when payments occur.

The setting corresponds to a lifecycle investment scheme aimed at planning for retirement in the absence of longevity risk, which means that we have a fixed time horizon *T*, which does not depend on the investor's survival. Therefore, no mortality projection needs to be assumed. We also have the hypothesis of no inflation or financial interest rate. We will consider the risk-free rate to be the basis and therefore we will fix it equal to zero. This allows us to concentrate on the pure structure of the investment strategy and savings management.

We assume that the market consists of a risky asset, whose value evolves as a geometric Brownian motion with drift (excess return) equal to *α* and diffusion coefficient (volatility) equal to *σ* as well as a riskless asset, which has a constant return of zero. In other words, we are carrying out the analysis net of risk-free interest rates. This ensures that any positive amount of money left in the fund at time *T* represents a gain in comparison with investing solely in the riskless asset throughout the time period, whereas a negative amount at time *T* represents a loss.

Define *C*(*t*) to be the net sum of all payment and withdrawal transactions by time *t* so that *C*(*t*) changes only by means of jumps, equal to

(10) $$\begin{array}{c} \Delta C\left(t\right)=\{\begin{array}{cc} +a & \text{for}\;t=0,1,\ldots ,T/2-1\;\\ -a & \text{for}\;t=T/2,T/2+1,\ldots ,T-1\;. \end{array} \end{array}$$

The dynamics of *X*(*t*), which is the total wealth accumulated at time *t*, are given by

(11) $$\begin{array}{c} dX\left(t\right)=\alpha \pi \left(t\right)X\left(t\right)dt+\sigma \pi \left(t\right)X\left(t\right)dW\left(t\right)+dC\left(t\right), \end{array}$$

where *W*(*t*) is a standard Brownian motion and *π*(*t*) is the proportion of *X*(*t*) invested in stocks.

The investor can, at any time *t* ∈ [0, *T*), choose the proportion *π*(*t*) of the total wealth *X*(*t*) to invest in the risky asset. This is the more general setting where we believe in a continuous rebalancing assumption, meaning that at any time *t*, the desired proportion of wealth invested in the risky asset can be chosen and is available in the market. This assumption is also known as dynamic allocation.

We concentrate on *X*(*T*), which is the so-called terminal wealth or the resulting outcome of a lifelong saving/consumption plan (The model is pretty restrictive and stylized; the income/consumption streams as well as the time horizon are deterministic. However, the main optimality result is not dependent on the nature of the payment stream, as long as the timings of the payments are deterministic. The only reason for specifying a particular form for the payment stream is so that the simulations for comparisons can be carried out in the performance illustrations).

In this setting, it is easily shown (see Appendix A) that the problem of maximizing the expected utility of terminal wealth for the utility function *u*_*γ*,*K*_,

(12) $$\begin{array}{c} \underset{\pi }{\max}\;𝔼\left[\frac{1}{\gamma }{\left(X\left(T\right)+K\right)}^{\gamma }\right], \end{array}$$

is solved by a strategy which invests an amount in the risky asset at time *t* equal to

(13) $$\begin{array}{c} \pi \left(t\right)X\left(t\right)=A\left(K+X\left(t\right)+g\left(t\right)\right), \end{array}$$

where *g*(*t*) = ∑_*l*=*t*_^*T*^Δ*C*(*l*) indicates the future payment stream that for sure must be taken into account to measure wealth at any time *t*, because it is a compromised payment/refund structure. In a continuous time payment stream, we would have *g*(*t*) = ∫_*t*_^*T*^ *dC*(*s*). A simple version of this particular optimisation problem was already solved by [6] with no consumption process *C*(*t*).

Note that strategy (13) consist in investing *A* times the accumulated wealth, where the leverage corresponds to *A* = (*α*/*σ* ^2^(1 − *γ*)), which is proportional to the inverse of (1 − *γ*), a parameter associated with risk aversion. This optimal strategy is similar to a constant proportion portfolio insurance (CPPI) strategy with multiplier *A*. The CPPI strategy guarantees a minimum level of wealth at some specified horizon, −*K* < *X*(*T*), and the exposure to the risky asset is a multiplier, *A*, times the cushion. In our problem, the cushion is the difference between wealth at time *t*, which is equal to *X*(*t*) + *g*(*t*), and a floor, which for us is not a given proportion of wealth as usual, but it is rather equal to −*K*.

This strategy gives rise to the identity:

(14) $$\begin{array}{c} X\left(t\right)+g\left(t\right)+K=Z\left(t\right), \end{array}$$

where *Z*(*t*) is a geometric Brownian motion of the form:

(15) $$\begin{array}{c} Z\left(t\right)=Z\left(0\right)\exp\left\{\left(\alpha A-\frac{1}{2}\sigma^{2}A^{2}\right)t+A\sigma W\left(t\right)\right\}, \end{array}$$

with *W* being a standard Brownian motion and *A* = *α*/(*σ* ^2^(1 − *γ*)). Further details can be found in [51]. The positivity of the geometric Brownian motion, together with the fact that *g*(*T*) = 0, proves that our lower bound for *X*(*T*) is effectively −*K*.

### 3.1. The Limiting Strategy

As we allow both *K* → *∞* and *γ* → −*∞* in such a way that *K* = Λ(1 − *γ*), the amount invested in the risky asset according to (13) converges to *α*Λ/*σ* ^2^. In other words, when taking the limit in (13), we see that the limiting optimal strategy is one in which a constant amount is invested in the risky asset. Additionally, we can prove this limiting optimal result from a direct optimisation of the exponential utility.

**Proposition 1**

The optimal strategy under the exponential utility $\overset{-}{u}$ is to invest a constant amount *α*Λ/*σ* ^2^ in the risky asset.

The proof of the CARA optimality can be found in Appendix B.

The consequence of Proposition 1 is that the optimal strategy for the limiting utility function is the limit of the optimal strategies, something which is not guaranteed to be the case in all optimisation problems.

If the limiting strategy is used, we have

(16) $$\begin{array}{c} dX\left(t\right)=\frac{\alpha^{2}\Lambda }{\sigma^{2}}\;dt+\frac{\alpha \Lambda }{\sigma }\;dW\left(t\right), \end{array}$$

resulting in the identity

(17) $$\begin{array}{c} X\left(t\right)=\frac{\alpha^{2}\Lambda }{\sigma^{2}}t+\frac{\alpha \Lambda }{\sigma }W\left(t\right). \end{array}$$

The limiting strategy has many interesting properties. For instance, the distribution of terminal wealth is easily obtained using the fact that *W* is a standard Brownian motion. As a consequence, typical downside risk measures of the terminal wealth distribution can be obtained analytically. This particular feature makes the limiting strategy very interesting from the point of view of investigating its performance compared to other standard product insurance investment strategies. Moreover, since we will concentrate on the yearly returns that provide exactly the median terminal wealth value for this strategy or internal return rates, we will explore how to approximate IRRs in a one-step formula. This will indeed speed up computations that are usually rather time consuming when addressing the performance of sophisticated investment strategies, especially if outcomes are path dependent.

## 4. Performance Measurement Methodology

In this section we illustrate the limiting strategy and compare it to other popular schemes, such as holding a constant proportion invested in stocks. We follow the performance measurement methodology of [1] when analysing life cycle investments, and in particular, we study the stochastic behaviour of terminal wealth distribution.

The performance measurement methodology is a tool which allows to compare products with different risks. To evaluate an investment plan, we search for its *equivalent benchmark strategy*. We consider two strategies *equivalent* if they have the same downside risk.

We have chosen to work with the expected shortfall as downside risk measure. Sometimes it is referred to as conditional tail expectation or tail value at risk. We denote expected shortfall (See more information on risk measures at [52].) with tolerance *θ* as ES_*θ*_ and define

(18) $$\begin{array}{c} \text{E}{\text{S}}_{\theta }=𝔼\left[X\left(T\right)\;|\;X\left(T\right)<v_{\theta }\right], \end{array}$$

where *v*_*θ*_ is the value at risk with the same tolerance, that is, the (1 − *θ*)% quantile of the distribution of *X*(*T*):

(19) $$\begin{array}{c} \mathbb{P}\left[X\left(T\right)>v_{\theta }\right]=\theta . \end{array}$$

The tolerance level is fixed at 95%. The *benchmark* strategy is often called the trivial strategy with a constant stock proportion *π* ^*b*^ for all *t* ∈ [0, *T*].

For any investment plan, and for its equivalent strategy, the internal interest rate (*r*_int⁡_) is calculated; that is,

(20) $$\begin{array}{c} \sum_{t=0}^{T-1}\Delta C\left(t\right){\left(1+r_{\mathrm{int}}\right)}^{T-t}-X_{m}\left(T\right)=0, \end{array}$$

where *X*_*m*_(*T*) is the median of the final wealth distribution; that is, *ℙ*(*X*(*T*) > *X*_*m*_(*T*)) = 0.5. Intuitively, a higher median is associated with a higher internal interest rate. The internal interest rate is the exact yearly rate of return that would lead to the median final wealth *X*_*m*_(*T*) given the structure of payments and withdrawals from initial wealth.

The difference between the internal interest rate of a CPPI (or any other) strategy *r*_int⁡_^*p*^ and that of the benchmark strategy *r*_int⁡_^*p*^, is called the yearly financial gain (whenever positive) or loss (whenever negative). The difference indicates whether or not the plan beats its risk-equivalent benchmark.

### 4.1. Calculation of the Expected Shortfall for the CPPI Strategy

From (14) and *g*(*T*) = 0, it can be proved (see Appendix C) that the value at risk at the *θ*th percentile for the CPPI strategy is given by

(21) $$\begin{array}{c} v_{\theta }=K\left\{\exp\left(\left(\alpha A-\frac{1}{2}A^{2}\sigma^{2}\right)T+\Phi^{-1}\left(1-\theta \right)A\sigma \sqrt{T}\right)-1\right\}, \end{array}$$

where Φ represents the standard Normal distribution function.

The expected shortfall is given by

(22) $$\begin{array}{c} {\text{ES}}_{\theta }=-K+\frac{1}{1-\theta }Ke^{\alpha AT}\Phi \left(\Phi^{-1}\left(1-\theta \right)-A\sigma \sqrt{T}\right), \end{array}$$

as proved in Appendix C.

This means that, if ES_*θ*_^*b*^ for the benchmark strategy *π* ^*b*^ is known, then the value of *K*_*b*_ which gives an equivalent expected shortfall is

(23) $$\begin{array}{c} K_{b}=\frac{{\text{ES}}_{\theta }^{b}}{-1+{\left(1-\theta \right)}^{-1}e^{\alpha AT}\Phi \left(\Phi^{-1}\left(1-\theta \right)-A\sigma \sqrt{T}\right)}. \end{array}$$

We will call *K*_*b*_/ES_*θ*_^*b*^ the * factor* that relates the maximum possible loss in the CPPI strategy to the expected shortfall of the benchmark. This means that one can compare the maximum possible loss to the expected loss of the benchmark product beyond some value at risk with a given tolerance.

### 4.2. Median Terminal Value for the Limiting Strategy

The median value of *X*(*T*) corresponds to the median value of *Z*(*T*), which in turn corresponds to the median value of *W*(*T*) which is 0, by symmetry. We therefore see that the median value of *X*(*T*) is

(24) $$\begin{array}{c} X_{m}\left(T\right)=-K+K\exp\left\{\left(\alpha A-\frac{1}{2}\sigma^{2}A^{2}\right)T\right\}. \end{array}$$

From (17), it clearly follows that the median value of *X*(*T*) when we use the limiting strategy of investing a constant amount in the risky asset is equal to

(25) $$\begin{array}{c} X_{m}\left(T\right)=\frac{\alpha^{2}\Lambda }{\sigma^{2}}T. \end{array}$$

This result indicates that in the limit, the only factor that alters the long-term median returns, given the market parameters, is the difference between initial and terminal time *T*. So, the obvious way to increase terminal wealth median returns is to widen life cycle as much as possible, thus making *T* large. This means that investors aiming at an optimal lifelong investment strategy should not delay the decision to start the savings phase.

### 4.3. Internal Rate of Return

The internal rate of return or internal interest rate is the value *r* which makes the discounted value of the payment stream *C*(*t*) equal to 0. In this simple situation, it means that

(26) $$\begin{array}{c} \sum_{s=0}^{T/2-1}a{\left(1+r\right)}^{T-s}-\sum_{s=T/2}^{T-1}a{\left(1+r\right)}^{T-s}-X\left(T\right)=0, \end{array}$$

which is equivalent to

(27) $$\begin{array}{c} a{\left({\left(1+r\right)}^{T/2}-1\right)}^{2}=\left(1-{\left(1+r\right)}^{-1}\right)X\left(T\right), \end{array}$$

as proved in Appendix D.

This equation defines *X*(*T*)/*a* in terms of *r* and the payment stream mechanism considered in the previous section; its inverse is the definition of *r* as a function of *X*(*T*)/*a*.

**Proposition 2**

For any given terminal wealth distribution *X*(*T*) and consumption as in (10), *X*(*T*)/*a* is an increasing function of *r* over the range *r* > 0.

The proof of Proposition 2 can be found in Appendix D. Moreover, it immediately follows from Proposition 2 that the median value of *r* corresponds to the median value of *X*(*T*).

Finally, as shown in Appendix E, the internal interest rate can be approximated by

(28) $$\begin{array}{c} r\approx \frac{1}{T}\left(-1+\sqrt{1+\frac{8X_{m}\left(T\right)}{cT}}\right). \end{array}$$

### 4.4. A Benchmark Strategy Providing an Equivalent Internal Rate of Return

All our performance evaluations will be connected to a benchmark strategy. We fix a simple, straightforward benchmark strategy. We have chosen to work with the investment that implies constant relative risk aversion, while wealth can fall below zero. This benchmark strategy implies that the investment is designed so that a constant proportion *π* ^*b*^ of the wealth is exposed to the risky assets at any time *t*. We can then write an expression for terminal wealth, so that *X*(*T*) is given by

(29) X(T)=a∑s=0T/2−1e(απb−(1/2)σ2πb2)(T−s)+σπb(W(T)−W(s)) −a∑s=T/2T−1e(απb−(1/2)σ2πb2)(T−s)+σπb(W(T)−W(s)).

This enables us to calculate its expectation;

(30) 𝔼[X(T)]=a∑s=0T/2−1eαπb(T−s)−a∑s=T/2T−1eαπb(T−s)=aeαπb(eαπbT/2−1)2eαπb−1.

This would correspond to an internal rate of return of *r* = *e* ^*απ*^*b*^^ − 1. Looking at it in another way, the value of *π* ^*b*^ required to achieve an internal rate of return equal to *r* would be *π* ^*b*^ = *α* ^−1^log⁡(1 + *r*).

The positive skewness of the distribution of *X*(*T*) means that the value of *π* ^*b*^ calculated by this formula will always be an underestimate of the true value of *π* ^*b*^ required to achieve this return. Indeed, it may be the case that there is *no* value of *π* ^*b*^ which gives an equivalent median return at this level.

## 5. Illustration

In our illustration, we are calculating the median of the final wealth, rather than the mean, and the corresponding internal interest rate based on the simulated distribution of the final wealth.

### 5.1. The Strategies and the Parameters

We set a time horizon of *T* = 60 years. We use *a* = 10 in our payment/consumption stream process. The risk-free rate of interest is set equal to zero because we are only interested in seeing the return that exceeds the risk-free rate. This obviously affects the choice of the excess interest rate return, *α*.

The constant proportion portfolio insurance (CPPI) strategy with a certain multiplier *A* guarantees a minimum level of wealth at some specified horizon. The investment mechanism is to invest *A* times *X*(*t*) + *g*(*t*) + *K* in risky assets, where *X*(*t*) is wealth at time *t*, *g*(*t*) is the discounted future payment stream, *K* is the guaranteed maximum possible loss, and *T* is the ultimate time horizon. In this model setup, the amount invested in risky assets at time *t* is equal to

(31) $$\begin{array}{c} \frac{\alpha }{\left(1-\gamma \right)\sigma^{2}}\left(X\left(t\right)+g\left(t\right)+K\right), \end{array}$$

and the terminal wealth after *T* = 60 years never falls below the guaranteed level −*K*.

In the benchmark strategy, a proportion of wealth is invested in stocks every period *t* and the wealth *X*(*t*) is a path-dependent process that we need to simulate in order to examine its terminal distribution after *T* = 60 years.

Setting the risk-free rate equal to zero has also been done in [1]. The choice of the risk-free rate does not affect the results, as has been investigated in [51]. With regard to the remaining parameters, we have estimated the yearly excess stock return to be equal to *α* = 3.43% and the volatility *σ* = 15.44%. Similar levels have recently been used by [1, 33, 53].

### 5.2. Values of the CPPI Leverage

Tables 1, 2, and 3 present the equivalent CPPI plan product for a variety of benchmark strategies corresponding to leverage values of *A* equal to 0.5, 1, and 2, respectively. Together with the value of *A* in each table, we also indicate the value of the corresponding factor from expression (23) that allows us to calculate the maximum possible loss *K* as a function of the downside risk measure, that is, the expected shortfall at the 95% level, of the benchmark strategy.

In each case, the first column indicates the percentage invested in stocks in the benchmark strategy. The second column corresponds to its expected shortfall at the 95% tolerance level, which is the left tail conditional expectation of wealth at *T*. The third column shows the value of *K* which would be required in our CPPI strategy to achieve the same median risk, derived from (23). The rest of columns are obtained by Monte Carlo simulation. Columns four and five are the internal interest rates for the benchmark and the CPPI strategies, respectively. Column six is the difference between internal interest rate returns. A positive sign indicates that the CPPI is better in comparison with the benchmark strategy with exactly the same expected shortfall. The last column shows the percentage which needs to be invested in stocks in a benchmark strategy plan in order to reach the same internal interest rate as the CPPI in the same row. If the sixth column is positive, then the final column is larger than the first column and the final column benchmark strategy would have more risk than the corresponding CPPI.

We see that the value of the factor is negative for the three tables and increases as *A* increases. This results in larger values of *K* as *A* decreases; for example, the value of *K* for the CPPI strategy equivalent to investing 10% in stocks is 42 for *A* = 0.5 and 13 for *A* = 2. We also observe that the CPPI strategy is better than the benchmark strategy for *A* no higher than 1. Moreover, in several cases the CPPI strategy is considerably outperforming the benchmark strategy; for example, the yearly financial gain in terms of internal interest rate is higher than 0.2% for *A* = 0.5 and *A* = 1, when compared to the benchmark strategy, and increases as the percentage invested in stocks increases. It reaches the value 1.10% when 100% is invested in stocks and *A* = 0.5. For *A* = 2, the CPPI plan underperforms the trivial strategy when the constant percentage invested in stocks is lower than 50% and performs equally at the level of 50%. This means that for *A* = 2 investing 50% in stocks in the benchmark strategy is equivalent to fixing the value of *K* = 83 in the CPPI strategy, both in terms of risk and performance as measured by the internal interest rate. For *A* lower than 2, there is no such equivalent investment in stocks compared to the CPPI plan. Finally, we also see that for *A* = 2 and a percentage higher than 50% invested in stocks, the CPPI plan again outperforms the trivial strategy but the financial gain is not so high as for lower values of *A*. In this sense, it would be interesting to find analytically a critical value of *A* for which the CPPI plan underperforms the trivial strategy.

In conclusion, in order to improve the typical fixed proportion invested in stocks that is a very common lifetime investment strategy, one should lower leverage *A*, which also implies raising *K* in the CPPI strategy. Recall that, as *A* is proportional to the reciprocal of the coefficient of risk aversion, lowering *A* is equivalent to increasing risk aversion.

In summary, our results show that whenever leverage *A* decreases, and we find the risk-adjusted equivalent to the benchmark strategy for a fixed *π* ^*b*^, then the maximum possible loss *K* increases. This indicates that, indeed, there exists a balance between risk aversion and the CPPI multiple. Moreover, CPPI is better than the trivial strategy for *A* ≤ 1 if we look at the yearly internal rates of return difference, but notably the performance of CPPI becomes much better than the benchmark when *A* diminishes as we approach the limiting optimal strategy. In several cases, the CPPI is quite considerably outperforming the benchmark strategy.

### 5.3. Graphical Display When Varying A

**Figure 1:**
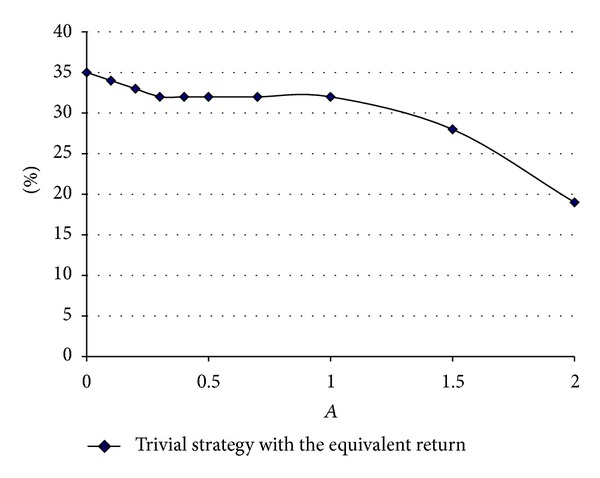
For different values of *A* (ranging from 0 to 2) dots indicate the equivalent proportion of wealth invested in stocks for the benchmark strategy which has the same median return as the CPPI which is exactly risk-equivalent to *π* ^*b*^ = 20%.

**Figure 2:**
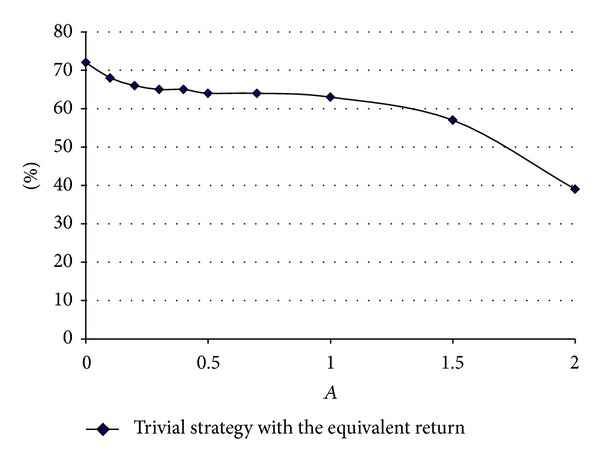
For different values of *A* (ranging from 0 to 2) dots indicate the equivalent proportion of wealth invested in stocks for the benchmark strategy which has the same median return as the CPPI which is exactly risk-equivalent to *π* ^*b*^ = 40%.

Figure 1 shows the proportion invested in stocks that the benchmark strategy requires in order to have the same median return as the equivalent CPPI plan that has the same risk as the benchmark that invests 20% in stocks. We study variations of CPPI as a function of *A*. We can see that it increases as *A* gets close to zero and reaches a value around 35%. We also observe that it diminishes after *A* = 1.  Figure 2 shows the same graph but for an initial level proportion of 40% in stocks. Again, we see that it increases for small values of *A* and it is around 70% for *A* close to zero and again diminishes after *A* = 1.

Since *A* is proportional to the reciprocal of risk aversion, these figures mean that, as the multiplier goes to zero, risk aversion increases, and as risk aversion is very large, the CPPI can allow the maximum possible loss amount *K* to increase. So, the CPPI strategy can produce returns that can be about the same as those obtained with a constant proportional strategy, but using *A* = 2.

## 6. Discussion and Conclusion

As a result of our investigation, we conclude that an investor should put at risk “whatever he can afford to lose,” *K*, and not just a positive cushion as in standard CPPI products. The investment should be leveraged with some constant *A*, and then in the limit, the resulting constant amount should be invested in the risky asset.

If the investor decides that he does not want to lose anything (compared to the short interest rate) then he cannot gain either, so that case is trivial. So, let us say that if a lifelong investor is not afraid of losing up to *K*, then the optimal strategy is to invest *A* times the accumulated wealth; that is, *A*∗(*K* + gains), where *A* is proportional to the inverse of the power of the utility function, that is, risk aversion. This is exactly the principle of CPPI strategies.

In one particular limit, if we let *A* and *K* act together increasing risk aversion and *K* to infinity, then we can reach the limiting optimal strategy that corresponds unsurprisingly to the constant absolute risk utility function.

This limiting optimality is a beautiful result, that has probably escaped the analysis because of the difficulties arising when studying lifetime investment strategies. The literature has mostly overlooked the fact that the floor that is used to talk about the investment cushion could be fixed below zero, in order to accept losses.

We have aimed at showing the inherent interaction between risk aversion and maximum possible loss, a topic that has not been treated in such a way before and that can perhaps explain why risk aversion is so difficult to communicate or even measure in real life.

We conclude that absolute relative risk aversion might be worth considering for long term savings.

We claim that the optimal strategy for long-term investors is to keep a constant amount invested in the risky assets throughout the whole investment horizon. The constant amount is equal to the product of the maximum possible loss times a multiple, which we can also call a leverage. Unlike other popular products, the optimal allocation does not need to be recalculated based on the performance of the markets. Thus, dynamic investment allocation is automatic and simple for our proposed strategy. Finally, and most importantly, it turns out that in the limit of our suitable combination of maximum possible loss and infinite risk aversion, wealth and accumulated returns do not determine the definition of the constant amount optimal strategy.

Our recommendation is not delaying or postponing decisions on lifetime savings/pension plans, because arising from the optimal limiting strategy of having a large constant amount invested in the risky assets combined with a large risk aversion, it follows that the longer the investment period, the larger the expected returns at the terminal time.

## Figures and Tables

**Table 1:** Equivalence between lifetime benchmark investment strategy (a fixed percentage π^*b*^ is invested in stocks) and the CPPI (fixed *K*). We set *T* = 60, μ = 3.43%, and σ = 15.44%. Here, we consider *A* = 0.5, so, the factor derived from (23) is equal to −3.255.

π^*b*^	ES (95%)	*K*	*r*_int⁡_^*b*^	*r*_int⁡_^*p*^	*r*_int⁡_^*p*^ − *r*_int⁡_^*b*^	Equivalent π^*b*^
10%	−12.82	42	0.33%	0.53%	0.20%	16%
20%	−27.34	89	0.64%	0.98%	0.34%	32%
30%	−43.43	141	0.93%	1.37%	0.44%	48%
40%	−61.03	199	1.19%	1.73%	0.54%	64%
50%	−80.12	261	1.43%	2.04%	0.62%	82%
60%	−100.73	328	1.64%	2.34%	0.69%	107%
70%	−123.16	401	1.84%	2.61%	0.77%	∗
80%	−147.67	481	2.01%	2.87%	0.86%	∗
90%	−174.85	569	2.16%	3.12%	0.97%	∗
100%	−205.36	668	2.27%	3.37%	1.10%	∗

*The maximum internal interest rate for the bechmarks is 2.5014% for 152% invested in stocks.

**Table 2:** Equivalence between lifetime benchmark investment strategy (a fixed percentage π^*b*^ is invested in stocks) and the CPPI (fixed *K*). We set *T* = 60, μ = 3.43%, and σ = 15.44%. Here, we consider *A* = 1.0, so, the factor derived from (23) is equal to −1.532.

π^*b*^	ES (95%)	*K*	*r*_int⁡_^*b*^	*r*_int⁡_^*p*^	*r*_int⁡_^*p*^ − *r*_int⁡_^*b*^	Equivalent π^*b*^
10%	−12.82	20	0.33%	0.53%	0.20%	16%
20%	−27.34	42	0.64%	0.97%	0.33%	32%
30%	−43.43	67	0.93%	1.37%	0.44%	47%
40%	−61.03	93	1.19%	1.70%	0.51%	63%
50%	−80.12	123	1.43%	2.03%	0.60%	81%
60%	−100.73	154	1.64%	2.31%	0.67%	104%
70%	−123.16	189	1.84%	2.59%	0.76%	∗
80%	−147.67	226	2.01%	2.85%	0.84%	∗
90%	−174.85	268	2.16%	3.10%	0.95%	∗
100%	−205.36	315	2.27%	3.35%	1.08%	∗

*The maximum internal interest rate for the bechmarks is 2.5014% for 152% invested in stocks.

**Table 3:** Equivalence between lifetime benchmark investment strategy (a fixed percentage π^*b*^ is invested in stocks) and the CPPI (fixed *K*). We set *T* = 60, μ = 3.43%, and σ = 15.44%. Here, we consider *A* = 2.0, so, the factor derived from (23) is equal to −1.033.

π^*b*^	ES (95%)	*K*	*r*_int⁡_^*b*^	*r*_int⁡_^*p*^	*r*_int⁡_^*p*^ − *r*_int⁡_^*b*^	Equivalent π^*b*^
10%	−12.82	13	0.33%	0.32%	−0.02%	10%
20%	−27.34	28	0.64%	0.62%	−0.02%	19%
30%	−43.43	45	0.93%	0.91%	−0.02%	29%
40%	−61.03	63	1.19%	1.17%	−0.01%	39%
50%	−80.12	83	1.43%	1.43%	0.00%	50%
60%	−100.73	104	1.64%	1.66%	0.02%	61%
70%	−123.16	127	1.84%	1.89%	0.05%	73%
80%	−147.67	153	2.01%	2.11%	0.11%	87%
90%	−174.85	181	2.16%	2.33%	0.17%	106%
100%	−205.36	212	2.27%	2.54%	0.27%	∗

*The maximum internal interest rate for the bechmarks is 2.5014% for 152% invested in stocks.

## Appendices

### A. Optimal Strategy in the CRRA Case

This proof is an adapted version of the one presented by [6] and it is also been revised from [51].

The control problem of choosing a strategy *π* = *π*(*t*, *x*) to maximize, for each 0 ≤ *t* < *T* and for each *x*,

(A.1) 𝒥(t,x,π)=      def  𝔼[u(X(T)) ∣ X(t)=x  ,strategy  π  is  used],

is a familiar one and is treated by, for example, [54]. The ingredients of the problem are the utility function *u* and the dynamical process *X*(*t*), which in our case evolve according to the stochastic differential equation:

(A.2) $$\begin{array}{c} dX\left(t\right)=\alpha \pi \left(t\right)X\left(t\right)dt+\sigma \pi \left(t\right)X\left(t\right)dW\left(t\right)+dC\left(t\right), \end{array}$$

where *π*(*t*) represents for each time *t* the proportion of the total wealth which is invested in the risky asset.

The optimal value function *V*(*t*, *x*) is defined as

(A.3) $$\begin{array}{c} V\left(t,x\right)=\underset{\pi }{\sup}𝒥\left(t,x,\pi \right). \end{array}$$

The Hamilton-Jacobi-Bellman methodology (HJB) allows us to write the optimality equation in the form

(A.4) $$\begin{array}{c} \underset{\pi }{\sup}\left[V_{t}+\alpha \pi xV_{x}+\frac{1}{2}\pi^{2}\sigma^{2}x^{2}V_{xx}\right]=0, \end{array}$$

for each *t* such that Δ*C*(*t*) = 0, where *V*_*t*_, *V*_*x*_, and *V*_*xx*_ denote partial derivatives of *V*. If Δ*C*(*t*) ≠ 0, the corresponding requirement is

(A.5) $$\begin{array}{c} V\left(t-,x\right)=V\left(t,x+dC\left(t\right)\right). \end{array}$$

As a function of *π*, this is quadratic, so the maximum is achieved at

(A.6) $$\begin{array}{c} \hat{\pi }=-\frac{\alpha }{\sigma^{2}x}\cdot \frac{V_{x}}{V_{xx}}, \end{array}$$

as long as *V*_*xx*_ < 0 (a condition which needs to be checked separately for each application of the theory). Substituting this into the optimality equation gives

(A.7) $$\begin{array}{c} V_{t}-\frac{\alpha^{2}}{2\sigma^{2}}\;\frac{V_{x}^{2}}{V_{xx}}=0, \end{array}$$

where Δ*C*(*t*) = 0.

In this case the terminal utility function *u* is *u*(*x*) = *γ* ^−1^(*x* + *K*)^*γ*^. This prompts us to investigate solutions of the form

(A.8) $$\begin{array}{c} V\left(t,x\right)=\frac{1}{\gamma }p{\left(t\right)}^{1-\gamma }{\left(x+q\left(t\right)\right)}^{\gamma }, \end{array}$$

which satisfy the boundary condition

(A.9) $$\begin{array}{c} V\left(T,x\right)=u\left(x\right)=\frac{1}{\gamma }{\left(x+K\right)}^{\gamma }. \end{array}$$

We find that

(A.10) $$\begin{array}{c} V\left(t,x\right)=\frac{1}{\gamma }{\left(x+K+g\left(t\right)\right)}^{\gamma }\exp\left(\frac{\alpha \gamma }{2\sigma^{2}\left(1-\gamma \right)}\left(T-t\right)\right), \end{array}$$

where *g*(*t*) = ∫_*t*_^*T*^ *dC*(*s*), with corresponding strategy

(A.11) $$\begin{array}{c} \hat{\pi }\left(t,x\right)=\frac{\alpha }{\sigma^{2}\left(1-\gamma \right)}\frac{x+K+g\left(t\right)}{x}. \end{array}$$

It is elementary to verify that *V*_*xx*_ < 0 when *γ* < 1.

As we have found a strategy $\hat{\pi }$ whose value function *V* satisfies both the optimality equation and the boundary condition, the verification theorem in [54] allows us to conclude that the strategy is optimal.

The resulting optimal strategy, characterised by the form $\hat{\pi }(t)X(t)=A(K+X(t)+g(t))$ is known as the constant proportion portfolio insurance (CPPI) strategy with multiplier *A*. The CPPI strategy guarantees a minimum level of wealth at some specified horizon, *X*(*T*)>−*K*.

### B. Optimal Strategy in the CARA Case

This investigation proceeds as in Appendix A, except that now the utility function is *u*(*x*) = −Λ*e* ^−*x*/Λ^.

We seek a solution of the form

(B.1) $$\begin{array}{c} V\left(t,x\right)=-J\left(t\right)\exp\left(-\frac{x}{\Lambda }\right). \end{array}$$

Then

(B.2) $$\begin{array}{c} V_{t}=-J^{\prime }\left(t\right)\exp\left(-\frac{x}{\Lambda }\right),\\ V_{x}=\frac{J\left(t\right)}{\Lambda }\exp\left(-\frac{x}{\Lambda }\right),\\ V_{xx}=-\frac{J\left(t\right)}{\Lambda^{2}}\exp\left(-\frac{x}{\Lambda }\right). \end{array}$$

For this to be a solution of (A.7) at times when Δ*C*(*t*) ≠ 0, we must have

(B.3) $$\begin{array}{c} \left[J^{\prime }\left(t\right)-\frac{\alpha^{2}}{2\sigma^{2}}J\left(t\right)\right]\exp\left(-\frac{x}{\Lambda }\right)=0, \end{array}$$

which has solution proportional to exp⁡(−(*α* ^2^/2*σ* ^2^)(*T* − *t*)). The behaviour at the times when Δ*C*(*t*) ≠ 0 is accounted for by a factor exp⁡(−(1/Λ)∫_*t*_^*T*^ *dC*(*s*)) = exp⁡(−*g*(*t*)/Λ). Along with the boundary condition *J*(*T*) = Λ, the solution is therefore

(B.4) $$\begin{array}{c} V\left(t,x\right)=-\Lambda \exp\left(-\frac{1}{\Lambda }\left[x+g\left(t\right)\right]-\frac{\alpha^{2}}{2\sigma^{2}}\left(T-t\right)\right),\\ x\hat{\pi }\left(t,x\right)=\frac{\alpha }{\sigma^{2}}\Lambda . \end{array}$$

Again, the verification theorem (see, e.g., [54]) enables us to deduce that this strategy is optimal.

### C. Calculation of the Expected Shortfall for the CPPI Strategy

The value at risk at the *θ*th percentile for the CPPI strategy is the value *v*_*θ*_ such that

(C.1) (1−θ) =ℙ[X(T)<vθ] =ℙ[Z(T)<vθ+K] =ℙ[W(T)T<1AσT×[log⁡(vθ+K)−log⁡(Z(0))−(αA−12A2σ2)T]].

Since $W(T)/\sqrt{T}$ is a standard Normal random variable, we can work out the value of *v*_*θ*_ explicitly in terms of the other parameters, noting that *Z*(0) = *X*(0−) + *g*(0−) + *K* = *K*:

(C.2) $$\begin{array}{c} v_{\theta }=K\left\{\exp\left(\left(\alpha A-\frac{1}{2}A^{2}\sigma^{2}\right)T+\Phi^{-1}\left(1-\theta \right)A\sigma \sqrt{T}\right)-1\right\}, \end{array}$$

where Φ represents the standard Normal distribution function.

The conditional tail expectation corresponding to a left-tail probability of (1 − *θ*)% is given by

(C.3) 𝔼[X(T) ∣ X(T)<vθ] =𝔼[−K+Kexp⁡((αA−12A2σ2)T)×eAσTY ∣ X(T)<vθ] =−K+Kexp⁡((αA−12A2σ2)T)  ×𝔼[eAσTY ∣ Y<Φ−1(1−θ)].

In this expression, *Y* stands for the ratio $W(T)/\sqrt{T}$, which we know to have a standard Normal distribution.

By definition, *ℙ*(*Y* < Φ^−1^(1 − *θ*)) = (1 − *θ*). We may therefore write the expected shortfall as

(C.4)  ESθ=−K+11−θKexp⁡((αA−12A2σ2)T) ×∫−∞Φ−1(1−θ)eAσTy×12πexp⁡(−y22)dy=−K+11−θKexp⁡((αA−12A2σ2)T) ×∫−∞Φ−1(1−θ)eAσTy12πexp⁡(−y22)=−K+11−θKexp⁡(αAT) ×∫−∞Φ−1(1−θ)12πexp⁡(−12(y−AσT)2)=−K+11−θKeαATΦ(Φ−1(1−θ)−AσT).

### D. Proof of Proposition 2

We have to prove that

(D.1) $$\begin{array}{c} \frac{1+r}{r}{\left({\left(1+r\right)}^{T/2}-1\right)}^{2} \end{array}$$

is increasing in *r* for *r* > 0. Proving that its logarithm is increasing will be sufficient. Differentiating the logarithm gives

(D.2) $$\begin{array}{c} \frac{1}{1+r}-\frac{1}{r}+T\frac{{\left(1+r\right)}^{T/2-1}}{{\left(1+r\right)}^{T/2}-1}, \end{array}$$

which can also be written as

(D.3) $$\begin{array}{c} \frac{1+\left(rT-1\right){\left(1+r\right)}^{T/2}}{r\left(1+r\right)\left({\left(1+r\right)}^{T/2}-1\right)\;}. \end{array}$$

The denominator is positive for *r* > 0, as the first, second, and third terms are positive. As for the numerator, it is an increasing function of *r*, since it has derivative (1/2)*T*(1 + *r*)^(*T*/2−1)^(1 + (*T* + 2)*r*), and its value at *r* = 0 is *T* > 0.

### E. Approximating the Internal Rate of Return

The internal rate of return, or internal interest rate, is the value *r* which makes the discounted value of the payment stream *C*(*t*) equal to 0. In this simple situation, it means that

(E.1) ∑s=0T/2−1a(1+r)T−s −∑s=T/2T−1a(1+r)T−s−X(T)=0.

Summing,

(E.2) $$\begin{array}{c} a\frac{{\left(1+r\right)}^{T}-{\left(1+r\right)}^{T/2}}{1-{\left(1+r\right)}^{-1}}-a\frac{{\left(1+r\right)}^{T/2}-1}{1-{\left(1+r\right)}^{-1}}=X\left(T\right), \end{array}$$

which is equivalent to

(E.3) $$\begin{array}{c} a{\left({\left(1+r\right)}^{T/2}-1\right)}^{2}=\left(1-{\left(1+r\right)}^{-1}\right)X\left(T\right). \end{array}$$

If *X*(*T*) = 0 it is clear that the internal rate of return is 0. When *X*(*T*) is not too large, we can use an asymptotic expansion to derive an approximate value for *r*:

(E.4) ((1+r)T/2−1)2 =(1+T2r+18T(T−2)r2+O(r3)−1)2 =T2r24(1+12(T−2)r+O(r2)).

Multiplying this by (1 − (1 + *r*)^−1^), we obtain

(E.5) $$\begin{array}{c} \frac{4X_{m}\left(T\right)}{cT^{2}}=r\left(1+\frac{1}{2}Tr+O\left(r^{2}\right)\right). \end{array}$$

This means that the value 4*X*_*m*_(*T*)/(*cT* ^2^) will provide a good approximation to the median value of *r* as long as *rT*/2 is quite close to 0, and in other cases we can use the quadratic derived from this expression to find a closer approximation,

(E.6) $$\begin{array}{c} r\approx \frac{1}{T}\left(-1+\sqrt{1+\frac{8X_{m}\left(T\right)}{cT}}\right). \end{array}$$
